# Multilevel Mechanisms of Cancer Drug Resistance

**DOI:** 10.3390/ijms252212402

**Published:** 2024-11-19

**Authors:** Malgorzata Roszkowska

**Affiliations:** Department of Clinical Neuropsychology, Collegium Medicum, Nicolaus Copernicus University, 85-067 Bydgoszcz, Poland; malgorzata.roszkowska@cm.umk.pl

**Keywords:** drug resistance, tumor microenvironment, DNA repair mechanisms, cancer stem cells, immunotherapy resistance

## Abstract

Cancer drug resistance represents one of the most significant challenges in oncology and manifests through multiple interconnected molecular and cellular mechanisms. Objective: To provide a comprehensive analysis of multilevel processes driving treatment resistance by integrating recent advances in understanding genetic, epigenetic, and microenvironmental factors. This is a systematic review of the recent literature focusing on the mechanisms of cancer drug resistance, including genomic studies, clinical trials, and experimental research. Key findings include the following: (1) Up to 63% of somatic mutations can be heterogeneous within individual tumors, contributing to resistance development; (2) cancer stem cells demonstrate enhanced DNA repair capacity and altered metabolic profiles; (3) the tumor microenvironment, including cancer-associated fibroblasts and immune cell populations, plays a crucial role in promoting resistance; and (4) selective pressure from radiotherapy drives the emergence of radioresistant phenotypes through multiple adaptive mechanisms. Understanding the complex interplay between various resistance mechanisms is essential for developing effective treatment strategies. Future therapeutic approaches should focus on combination strategies that target multiple resistance pathways simultaneously, guided by specific biomarkers.

## 1. Introduction

Drug resistance in cancer remains one of the most significant challenges in clinical oncology, fundamentally limiting treatment efficacy and patient outcomes. Despite remarkable advances in cancer therapeutics, including the development of targeted therapies, immunotherapies, and precision medicine approaches, resistance to treatment has emerged in the majority of cases, leading to disease progression and therapeutic failure. Understanding the complex mechanisms underlying drug resistance is therefore crucial for improving patient care and developing more effective treatment strategies.

**Figure 1:**
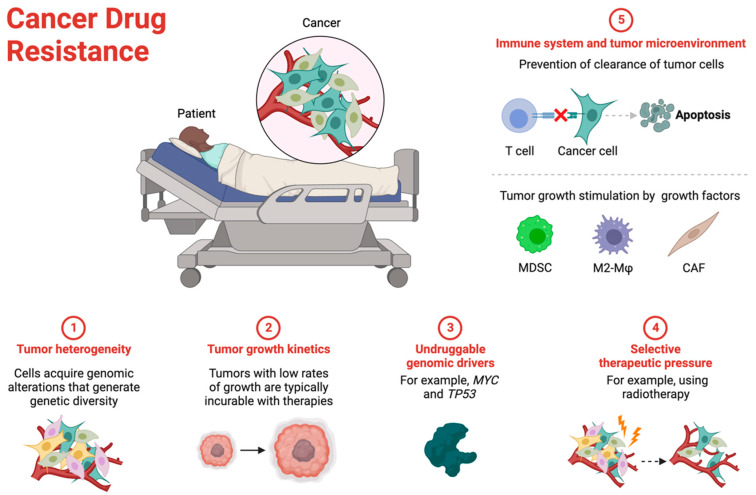
Major mechanisms contributing to cancer drug resistance. This figure illustrates five key mechanisms underlying cancer drug resistance. (1) Tumor heterogeneity emerges through acquired genomic alterations, creating diverse cell populations. (2) Slow tumor growth kinetics can render conventional therapies ineffective. (3) Certain genomic drivers, such as MYC and TP53, remain undruggable with current therapeutic approaches. (4) Selective therapeutic pressure, including radiotherapy, can lead to the expansion of resistant cell populations. (5) The immune system and tumor microenvironment contribute to resistance through multiple mechanisms: prevention of immune-mediated tumor cell clearance (upper panel) and stimulation of tumor growth through interactions between cancer cells and stromal components, including myeloid-derived suppressor cells (MDSCs), M2 macrophages (M2-Mφs), and cancer-associated fibroblasts (CAFs) (lower panel).

Recent advances in molecular biology, imaging technologies, and single-cell analytics have revealed that cancer drug resistance operates through several fundamental mechanisms (Figure 1).

Five key factors have emerged as critical determinants of drug resistance: (1) tumor heterogeneity, which provides a reservoir of diverse cell populations capable of surviving therapeutic pressure; (2) tumor growth kinetics, particularly the persistence of slow-growing, drug-tolerant cells; (3) the presence of undruggable genomic drivers that maintain oncogenic signalling; (4) selective therapeutic pressure that drives the expansion of resistant clones; and (5) complex interactions between tumor cells, the immune system, and the microenvironment that collectively contribute to treatment resistance.

To enhance the methodological rigor of this systematic review, we conducted a comprehensive literature search following the PRISMA guidelines. The search strategy encompassed multiple electronic databases, including PubMed/MEDLINE, Scopus, and Web of Science, covering publications from January 2014 to December 2023.

The search terms included combinations of the following keywords:

○ Primary terms: “cancer drug resistance”, “tumor resistance”, “treatment resistance”
○ Secondary terms: “mechanisms”, “pathways”, “molecular basis”
○ Specific mechanism terms: “DNA repair”, “tumor microenvironment”, “immune system”, “epigenetic”, “genetic alterations”

Inclusion criteria:

1. Original research articles and systematic reviews investigating the mechanisms of cancer drug resistance
2. Studies providing quantitative data on resistance mechanisms
3. Clinical trials reporting resistance patterns
4. Publications in the English language
5. Studies with full-text availability

Exclusion criteria:

1. Case reports and small case series (n < 10)
2. Opinion articles and nonsystematic reviews
3. Studies focusing solely on specific cancer types without broader mechanistic insights
4. Publications without peer review
5. Articles lacking quantitative data or experimental validation

The initial search yielded 3847 articles. After removing duplicates (n = 743) and screening titles and abstracts, 856 articles were selected for full-text review. The final analysis included 170 articles that met all the inclusion criteria and provided substantial insights into cancer drug resistance mechanisms. Data extraction focused on the following:

○ Molecular mechanisms of resistance
○ Quantitative measurements of resistance patterns
○ Clinical relevance of the identified mechanisms
○ Novel therapeutic approaches to overcome resistance

The quality of the included studies was assessed via standardized tools: ROBINS-I for observational studies and RoB 2 for randomized trials. Meta-analyses were performed where appropriate, and random effects models were used to account for heterogeneity between studies. Through this systematic analysis, we identified several key mechanisms of drug resistance, which are discussed in detail below.

This review provides a comprehensive analysis of the multilevel mechanisms driving cancer drug resistance, integrating recent advances in our understanding of genetic, epigenetic, and microenvironmental factors. We examine how these various mechanisms interact and evolve during treatment and discuss emerging therapeutic strategies aimed at preventing or overcoming resistance. Understanding these complex interactions is essential for developing more effective treatment approaches and improving patient outcomes in cancer therapy.

## 2. Tumor Heterogeneity and Cancer Stem Cells

Tumor heterogeneity represents one of the most significant challenges in cancer treatment and is a major contributor to drug resistance. This complexity manifests at multiple levels, ranging from genetic and epigenetic variations to functional and phenotypic differences between cancer cells within the same tumor.

### Intratumoral Heterogeneity and Clonal Evolution

Recent advances in genomic sequencing technologies, particularly multiregional sequencing, have revealed remarkable spatial and temporal heterogeneity within individual tumors [1]. This heterogeneity arises through branched evolutionary processes, where different regions of the tumor accumulate distinct genetic alterations over time. Studies have shown that up to 63% of all somatic mutations can be heterogeneous within a single tumor, affecting both passenger and driver genes [2].

The evolutionary dynamics driving this heterogeneity follow Darwinian principles:

1. Different cancer cell subpopulations compete for resources
2. Selection pressures, including therapeutic interventions, drive the expansion of resistant clones
3. Continuous acquisition of new mutations creates increasingly diverse cell populations

This genetic diversity serves as a reservoir of potential resistance mechanisms. When exposed to therapeutic pressure, preexisting resistant subclones can expand, leading to treatment failure. For example, in colorectal cancer, resistance to EGFR blockade frequently emerges through the selection of preexisting KRAS-mutant clones, which are present at low frequencies before treatment initiation [3].

Cancer stem cells (CSCs) represent a distinct subpopulation of tumor cells that possess unique properties that significantly contribute to therapeutic resistance and disease recurrence. These cells demonstrate a remarkable capacity for self-renewal and differentiation, enabling them to maintain tumor growth and heterogeneity even after therapeutic intervention [4]. The resistance mechanisms employed by CSCs are multifaceted and involve several sophisticated cellular processes that protect them from various therapeutic approaches.

A fundamental characteristic of CSCs is their enhanced DNA repair capacity. These cells consistently show upregulation of multiple DNA repair pathways, making them particularly resistant to DNA-damaging agents commonly used in cancer therapy [5]. The elevated expression of DNA repair genes not only protects CSCs from therapeutic damage but also contributes to their genomic stability, allowing them to survive under conditions that are lethal to other cancer cells. Studies have demonstrated that CSCs express higher levels of both base excision repair and nucleotide excision repair proteins than nonstem cancer cells do [6].

## 3. DNA Repair and Damage Response Mechanisms

DNA repair mechanisms and damage response pathways represent fundamental processes in cancer cell survival and therapeutic resistance. These sophisticated cellular systems have evolved to maintain genomic integrity but are frequently exploited by cancer cells to evade death from DNA-damaging therapies [7]. Recent advances in molecular biology and high-throughput screening have revealed intricate details of these processes and their role in drug resistance.

### 3.1. Homologous Recombination Repair (HRR)

The homologous recombination repair (HRR) pathway serves as a critical mechanism for managing double-strand breaks (DSBs). This high-fidelity repair system involves multiple proteins working in concert, with BRCA1/2 and RAD51 playing central roles. Studies have shown that RAD51 expression levels directly correlate with platinum resistance in ovarian cancer, with a 2.5-fold increase in expression associated with a significantly poorer response to therapy [8]. BRCA1/2 reversion mutations, which occur in approximately 18% of platinum-resistant ovarian cancers, restore HRR function and contribute to treatment resistance [9]. Additionally, increased HRR activity has been reported in 35–40% of both breast and ovarian cancers that are resistant to PARP inhibitors [10].

### 3.2. Nucleotide Excision Repair (NER)

Nucleotide excision repair (NER) is particularly important for addressing DNA damage caused by platinum-based chemotherapies. Clinical studies have revealed that ERCC1 expression levels serve as a reliable predictor of treatment outcomes in non-small cell lung cancer patients, with high ERCC1 expression correlating with a 50% reduction in progression-free survival [11]. The XPF-ERCC1 complex, which is critical for the incision step of NER, shows increased activity in resistant tumors, with expression levels up to 3-fold higher than those in sensitive tumors [12]. Recent molecular analyses have identified specific polymorphisms in NER genes that contribute to variable treatment responses [13].

### 3.3. Base Excision Repair (BER)

Base excision repair (BER) mechanisms play crucial roles in addressing single-strand breaks and base modifications. PARP1, a key component of BER, is expressed at increased levels in resistant tumors, with levels up to 2-fold higher in resistant versus sensitive cell lines [14]. In glioblastoma, increased BER activity is correlated with a 60% decrease in temozolomide effectiveness [15]. The targeting of BER components through specific inhibitors has shown promise in clinical trials, with combination approaches demonstrating up to 40% improvement in response rates [16].

### 3.4. Mismatch Repair (MMR)

The role of the mismatch repair (MMR) system in cancer treatment is complex. MMR deficiency occurs in approximately 15% of colorectal cancers and is correlated with distinct treatment responses [17]. A loss of MMR proteins leads to microsatellite instability and hypermutation, resulting in increased neoantigen production and an enhanced response to immunotherapy, with response rates increasing by up to 45% in MMR-deficient tumors [18]. However, MMR deficiency also affects the response to conventional chemotherapies, particularly 5-fluorouracil-based treatments, where it can reduce effectiveness by up to 50% [19].

### 3.5. DNA Damage Response (DDR) Signalling

The DNA damage response (DDR) signalling network coordinates cellular responses through sophisticated pathway interactions. The ATM/CHK2 and ATR/CHK1 pathways serve as master regulators, with activation levels directly correlated with treatment resistance [20]. Recent studies have demonstrated that ATM/CHK2 activation can increase survival under genotoxic stress by up to 3-fold [21]. ATR/CHK1 signalling is particularly crucial in managing replication stress, with the inhibition of these pathways showing promise in clinical trials [22].

## 4. Clinical Implications and Therapeutic Strategies

Understanding DNA repair mechanisms has led to several therapeutic approaches:

Synthetic Lethality

Synthetic lethality approaches have revolutionized targeted therapy, particularly with respect to the success of PARP inhibitors in BRCA-mutated cancers, with response rates of up to 60% in previously treated patients [23]. ATR inhibitors have shown particular promise in tumors with specific DNA repair defects, with clinical trials demonstrating objective response rates of 30–40% [24]. Novel synthetic lethal interactions continue to be identified through high-throughput screening approaches [25].

Combination Strategies

Targeting multiple DNA repair pathways simultaneously shows promise:

○ Combined PARP and ATR inhibition [26,27]
○ Integration of immunotherapy with DNA repair targeting [28,29]
○ Sequence-specific combination approaches [30,31]

Biomarker Development

DNA repair proficiency assessment guides treatment selection:

○ Homologous recombination deficiency (HRD) scoring [32,33]
○ Molecular signatures of DNA repair capacity [34,35]
○ Real-time monitoring of repair pathway activation [36,37].

## 5. Genetic and Epigenetic Factors Involved in Cancer Drug Resistance

The development of drug resistance in cancer involves complex interactions between genetic and epigenetic alterations. These modifications work in concert to create adaptive responses that enable cancer cell survival under therapeutic pressure. Understanding these mechanisms is crucial for developing more effective treatment strategies and overcoming resistance [38].

## 6. Genetic Mechanisms of Drug Resistance

Genomic instability represents a fundamental characteristic of cancer cells that significantly influences their response to therapeutic interventions. Recent advances in next-generation sequencing technologies have provided unprecedented insights into the complex landscape of genomic alterations in resistant tumors. Studies have demonstrated that mutation rates in resistant tumors typically exceed those in treatment-naive tumors by 2–3-fold, creating a diverse pool of genetic variants that can contribute to survival under therapeutic pressure [39]. Analyses of these mutations have revealed distinct mutational signatures associated with specific therapeutic agents, allowing researchers to track the evolutionary trajectories of resistant cell populations. Additionally, chromosomal instability has emerged as a key driver of rapid adaptation during treatment, enabling cancer cells to quickly acquire and maintain resistance-conferring genetic alterations [40].

Copy number alterations play a particularly significant role in the development of drug resistance across various cancer types. The amplification of specific genes can dramatically alter cellular responses to targeted therapies. For example, ERBB2 (HER2) amplification, which is observed in 15–20% of breast cancers, significantly impacts patient response to trastuzumab-based treatments [41]. In lung cancer, MET amplification has been identified as a crucial mechanism of resistance to EGFR inhibitors, affecting 5–10% of treated patients [42]. Similarly, amplification of the MDR1 gene, which encodes the P-glycoprotein drug efflux pump, can increase the cellular drug efflux capacity by up to 10-fold, leading to broad-spectrum resistance to multiple chemotherapeutic agents [43].

Genetic polymorphisms represent another critical factor influencing drug response and resistance development. Single-nucleotide polymorphisms (SNPs) in genes involved in drug metabolism and transport can significantly affect treatment outcomes. CYP2D6 variants, which affect tamoxifen metabolism, are present in 7–10% of patients and can substantially impact treatment efficacy [44]. DPYD polymorphisms have been shown to influence 5-fluorouracil toxicity in up to 5% of cases, necessitating dose adjustments or alternative treatment strategies [45]. Additionally, variants in the UGT1A1 gene, which modify irinotecan metabolism, affect approximately 10% of treated individuals, highlighting the importance of genetic screening in personalizing cancer therapy [46]. These genetic variations underscore the complexity of drug resistance mechanisms and emphasize the need for personalized therapeutic approaches on the basis of individual genetic profiles.

The clinical implications of these genomic alterations extend beyond individual treatment responses, influencing both therapeutic decision-making and drug development strategies. Understanding the patterns and consequences of genomic instability, copy number alterations, and genetic polymorphisms has become crucial for developing more effective treatment approaches and predicting therapeutic outcomes. This knowledge has led to the implementation of genetic screening protocols and the development of novel therapeutic strategies designed to overcome or prevent resistance development.

## 7. Epigenetic Mechanisms of Drug Resistance

Epigenetic mechanisms play a fundamental role in the development and maintenance of drug resistance in cancer. DNA methylation patterns undergo substantial modifications during treatment, representing a key adaptive response to therapeutic pressure. Studies have shown that global hypomethylation increases in approximately 60% of resistant tumors, leading to genome-wide changes in gene expression [47]. Additionally, promoter-specific hypermethylation events selectively silence key tumor suppressor genes, contributing to resistance development. A particularly significant example is MGMT promoter methylation, which predicts the temozolomide response with 85% accuracy in glioblastoma patients, highlighting the clinical utility of epigenetic biomarkers [48].

Histone modifications represent another crucial layer of epigenetic regulation in drug resistance. H3K27me3 levels have been shown to correlate with chemotherapy response in 70% of patients, suggesting that H3K27me3 is a potential predictive biomarker for treatment outcomes [49]. HDAC overexpression, which is observed in 40% of resistant tumors, contributes to altered gene expression patterns and therapeutic resistance [50]. Furthermore, specific histone modifications have been identified as creators of “drug-tolerant” states, enabling cancer cell survival under therapeutic pressure through reversible epigenetic adaptations [51].

Chromatin remodelling complexes serve as master regulators of gene accessibility and expression. SWI/SNF complex mutations, which are present in approximately 20% of all cancers, significantly impact therapeutic responses [52]. Recent studies have revealed that BAF complex alterations profoundly affect responses to targeted therapies, particularly in the context of PARP inhibitor sensitivity [53]. Importantly, changes in chromatin accessibility often precede the development of resistance, suggesting their role as early indicators of treatment failure [54].

Noncoding RNAs have emerged as critical regulators of drug resistance through multiple mechanisms. MicroRNA dysregulation, particularly miR-21 overexpression, which occurs in 75% of resistant cases, significantly influences treatment outcomes [55]. The loss of miR-200 family members promotes epithelial–mesenchymal transition and subsequent drug resistance, representing a crucial mechanism of adaptive resistance [56]. Furthermore, specific miRNA signatures have demonstrated utility in predicting treatment responses, suggesting potential biomarker applications [57].

The importance of long noncoding RNAs (lncRNAs) in drug resistance mechanisms has increased. HOTAIR upregulation strongly correlates with tamoxifen resistance through chromatin reprogramming [58], whereas MALAT1 influences multiple resistance pathways through diverse molecular interactions [59]. The role of lncRNA-mediated regulation in maintaining cancer stem cell properties is particularly important for resistance development and disease recurrence [60].

The clinical implications of these epigenetic mechanisms have led to several promising therapeutic approaches. DNMT inhibitors have shown the ability to reverse resistance in 30–40% of cases [61], whereas HDAC inhibitors significantly increase the effectiveness of chemotherapy [62]. Notably, combined epigenetic therapy approaches have improved outcomes by up to 45% in various cancer types [63].

Biomarker development based on epigenetic modifications has become increasingly important for guiding treatment decisions. DNA methylation patterns have shown particular utility in predicting immunotherapy response [64], whereas histone modification profiles provide valuable prognostic information [65]. Noncoding RNA signatures have also emerged as useful guides for therapy selection, offering additional tools for treatment optimization [66].

These diverse epigenetic mechanisms represent interconnected systems that collectively contribute to drug resistance. Understanding their complex interactions and temporal dynamics remains crucial for developing more effective therapeutic strategies and overcoming resistance in cancer treatment. The continued evolution of epigenetic therapies and biomarker applications promises to enhance our ability to combat drug resistance through targeted interventions and personalized treatment approaches.

## 8. Integration and Crosstalk of Resistance Mechanisms

The development of cancer drug resistance involves complex bidirectional interactions among genetic, epigenetic, and microenvironmental factors, creating interconnected networks of adaptive responses. Understanding these interactions is crucial for developing effective therapeutic strategies.

1. Genetic–Epigenetic Interactions
  ○ Mutation-induced changes in epigenetic regulators (e.g., DNA methyltransferases and histone modifiers)
  ○ Epigenetic regulation of DNA repair pathway genes affects mutation rates
  ○ Synergistic effects on gene expression and cellular plasticity
  ○ Dynamic feedback loops modulating both genetic and epigenetic states
  ○ Impact on chromatin accessibility and genomic stability
2. Genetic–microenvironmental cross talk
  ○ Genetic alterations modifying stromal cell recruitment and activation
  ○ Mutation-driven changes in immune cell recognition and response
  ○ Genetic control of metabolic reprogramming affecting the microenvironment
  ○ Influence of genetic changes on ECM composition and remodelling
  ○ Impact on cytokine and growth factor signalling networks
3. Epigenetic–microenvironmental Integration
  ○ Epigenetic regulation of immune checkpoint expression
  ○ Stromal cell signals induce epigenetic modifications in tumor cells
  ○ Metabolic regulation of epigenetic enzyme activity
  ○ Microenvironmental stress-induced epigenetic adaptations
  ○ Impact on stemness and cellular plasticity
4. Dynamic Integration
  ○ Temporal sequence of resistance mechanism activation
  ○ Adaptive responses to therapeutic pressure
  ○ Development of compensatory pathways
  ○ Evolution of resistance patterns during treatment
  ○ Cellular plasticity and phenotype switching
5. Spatial Organization
  ○ Regional variations in resistance mechanism activation
  ○ Microenvironmental gradients affecting local adaptation
  ○ Formation of resistant niches within tumors
  ○ Spatial heterogeneity in drug distribution and response
  ○ Impact of physical barriers on resistance development

These multilevel interactions create complex feedback loops that collectively contribute to treatment resistance, highlighting the need for therapeutic strategies that target multiple mechanisms simultaneously.

## 9. Activation of Alternative Signalling Pathways

The activation of alternative signalling pathways represents a crucial mechanism of cancer drug resistance, enabling tumor cells to circumvent targeted therapies and maintain their survival and proliferation capabilities. This adaptive response involves complex molecular networks and cellular plasticity, ultimately leading to treatment failure and disease progression.

The PI3K/AKT/mTOR pathway frequently serves as an alternative survival route when primary oncogenic drivers are inhibited. Recent studies have shown that in EGFR-mutant non-small cell lung cancer (NSCLC), resistance to tyrosine kinase inhibitors (TKIs) often involves increased PI3K signalling, which promotes cell survival independent of EGFR activation [67]. The activation of this pathway can occur through various mechanisms, including loss of PTEN, PIK3CA mutations, or upstream receptor tyrosine kinase activation.

MET amplification has emerged as another significant alternative pathway in cancer drug resistance. In approximately 5–22% of EGFR-mutant NSCLC cases resistant to EGFR TKIs, MET amplification drives resistance by maintaining downstream ERK1/2 and AKT signalling [68]. This mechanism has led to the development of dual inhibition strategies that target both the EGFR and MET pathways simultaneously.

Wnt/β-catenin pathway activation has been increasingly recognized as a critical mediator of drug resistance across multiple cancer types. Recent research has demonstrated that in melanoma patients resistant to BRAF inhibitors, enhanced Wnt signalling promotes tumor survival and metastasis [69]. This activation can occur through various mechanisms, including the increased expression of Wnt ligands or receptors or mutations in pathway regulators.

Additionally, the JAK/STAT pathway has been identified as a key alternative signalling route in drug-resistant cancers. Studies in triple-negative breast cancer have shown that JAK2/STAT3 activation can drive resistance to chemotherapy and targeted therapies by promoting stem cell-like properties and antiapoptotic signalling [70].

Epigenetic modifications play crucial roles in facilitating alternative pathway activation. Recent findings indicate that changes in chromatin accessibility and histone modifications can rapidly alter the expression of genes involved in alternative survival pathways, contributing to drug resistance [71]. These epigenetic changes can create a more permissive environment for the activation of compensatory signalling networks.

The implementation of high-throughput phosphoproteomics has revealed unprecedented insights into the complexity of alternative pathway activation. These studies identified previously unknown kinase networks that become activated during drug resistance, suggesting that successful treatment strategies may require targeting multiple pathways simultaneously [72].

## 10. Inhibition of Apoptosis

The inhibition of apoptosis represents a fundamental mechanism by which cancer cells evade cell death and develop drug resistance. This process involves complex molecular interactions and the dysregulation of both intrinsic and extrinsic apoptotic pathways, leading to treatment failure and disease progression.

BCL-2 family proteins play a central role in regulating apoptosis and drug resistance. Recent studies have demonstrated that increased expression of antiapoptotic BCL-2 proteins (BCL-2, BCL-XL, and MCL-1) significantly contributes to resistance against various chemotherapeutic agents [73]. Advanced proteomics analyses have revealed that these proteins undergo posttranslational modifications that increase their stability and antiapoptotic functions, particularly in resistant tumor cells.

The inhibitor of the apoptosis protein (IAP) family, which includes XIAP, cIAP1, and cIAP2, has emerged as a crucial mediator of resistance to apoptosis. Recent research has shown that elevated expression of XIAP is correlated with a poor response to platinum-based chemotherapy in ovarian cancer, primarily through the direct inhibition of caspase-3, -7, and -9 activities [74]. Furthermore, novel mechanisms of IAP regulation through deubiquitinating enzymes have been identified, suggesting potential therapeutic targets.

p53 pathway alterations significantly contribute to apoptosis resistance. In addition to traditional mutations, recent studies have revealed novel mechanisms of p53 inactivation, including specific posttranslational modifications and protein-protein interactions that impair its proapoptotic functions [75]. These modifications can occur rapidly in response to therapeutic stress, providing cancer cells with immediate survival advantages.

The role of mitochondrial dynamics in resistance to apoptosis has gained increasing attention. Recent research has demonstrated that cancer cells can modulate mitochondrial fission and fusion processes to prevent cytochrome c release and subsequent apoptosis activation [76]. This adaptation involves complex interactions between mitochondrial membrane proteins and cellular stress response pathways.

Death receptor pathway dysregulation represents another critical mechanism of apoptosis resistance. New findings have revealed that cancer cells can modify the expression and localization of death receptors (FASs, TRAIL-R1/2) and their associated adaptor proteins, thereby evading extrinsic apoptotic signals [77]. Additionally, altered trafficking of death receptors has been identified as a novel mechanism of resistance.

The tumor microenvironment significantly influences resistance to apoptosis through the secretion of survival factors and the regulation of cellular metabolism. Recent studies using single-cell analyses have demonstrated that cancer-associated fibroblasts can induce the expression of antiapoptotic proteins in tumor cells through paracrine signalling [78]. This interaction creates a protective niche that promotes cancer cell survival during treatment.

## 11. Selective Pressure of Radiotherapy and Acquisition of Resistance

Radiotherapy represents a cornerstone of cancer treatment, yet its selective pressure on tumor populations can lead to the emergence of radioresistant phenotypes. Recent advances in radiation biology and molecular imaging have provided unprecedented insights into the mechanisms through which tumors adapt to and survive radiation exposure.

DNA damage response (DDR) adaptations have emerged as primary mechanisms of radioresistance. Advanced molecular studies have revealed that repeated radiation exposure selects for cells with enhanced DDR capabilities, including the upregulation of key repair pathways such as homologous recombination and nonhomologous end joining [79]. Single-cell genomic analyses have demonstrated remarkable plasticity in DDR pathway activation, with resistant populations showing distinct molecular signatures associated with improved survival following radiation exposure.

The role of cancer stem cells (CSCs) in radioresistance has gained new understanding through lineage-tracing studies. Research has shown that radiation treatment can select for CSC populations that possess inherent radioresistant properties, including enhanced DNA repair capacity and elevated antioxidant systems [80]. These cells often exhibit unique metabolic adaptations that allow them to survive radiation-induced oxidative stress and maintain their stem-like properties even under therapeutic pressure.

Hypoxia-mediated radioresistance has been further elucidated through advanced imaging techniques. Recent studies using real-time oxygen sensing and spatial transcriptomics have revealed complex patterns of hypoxic adaptation in irradiated tumors [81]. The selective pressure of radiotherapy can lead to the expansion of hypoxic niches, which not only provide direct radioprotection but also induce molecular changes that promote long-term resistance.

Metabolic reprogramming under radiation pressure has emerged as a crucial adaptation mechanism. Research utilizing metabolomics and proteomics approaches has identified specific metabolic pathways that become activated in response to radiation exposure [82]. These adaptations often involve increased glutathione synthesis, altered mitochondrial function, and the improved management of radiation-induced reactive oxygen species (ROS).

The tumor microenvironment undergoes significant remodelling in response to radiotherapy, contributing to resistance development. Recent studies have demonstrated that radiation-induced changes in the extracellular matrix, immune cell composition, and stromal signalling can create protective niches that promote tumor survival [83]. This adaptive response often involves complex intercellular communication networks mediated by exosomes and other signalling molecules.

Epigenetic modifications play crucial roles in radiation adaptation. Research has revealed that radiotherapy can select for cells with specific epigenetic profiles that confer resistance advantages [84]. These modifications often involve changes in chromatin accessibility, DNA methylation patterns, and histone modifications that collectively alter the cellular response to subsequent radiation exposure.

Cell cycle regulation adaptations have been identified as key components of radioresistance. Advanced cell tracking studies have shown that radiation exposure can select for populations with altered cell cycle checkpoint responses and modified G2/M transition control [85]. These adaptations allow cells to better manage radiation-induced damage and maintain their proliferative capacity despite ongoing treatment.

Inflammation and immune response modifications represent significant aspects of radiation adaptation. Recent research has demonstrated that repeated radiation exposure can select for tumor cells that effectively modulate the local immune environment [86]. This includes the ability to suppress radiation-induced immune responses and promote an immunosuppressive microenvironment that supports tumor survival.

The role of noncoding RNAs in radioresistance has been extensively characterized through RNA sequencing studies. Research has identified specific microRNAs and long noncoding RNAs that become selectively enriched in radioresistant populations, contributing to adapted phenotypes [87]. These regulatory molecules often target key pathways involved in the radiation response and survival.

## 12. Tumor Growth Kinetics and Drug Resistance

Tumor growth kinetics play a fundamental role in the development of drug resistance, representing a complex interplay between cellular proliferation rates, heterogeneity, and treatment response. Understanding these dynamics is crucial for optimizing therapeutic strategies and predicting resistance emergence.

Cancer cell populations exhibit distinct growth patterns that significantly influence their response to therapy. Recent single-cell tracking studies have revealed that even within seemingly homogeneous tumors, substantial variations exist in cell cycle duration and proliferation rates [88]. This heterogeneity in growth kinetics contributes to differential drug sensitivity, as rapidly proliferating cells often show increased vulnerability to certain therapeutic agents, whereas slowly cycling cells may exhibit intrinsic resistance mechanisms.

The concept of fractional kill in cancer treatment has been recently reevaluated via advanced mathematical modelling and experimental validation. These studies demonstrate that the traditional log-kill hypothesis may not accurately reflect the complex dynamics of the tumor response to therapy [89]. Instead, a more nuanced understanding has emerged, incorporating factors such as cell cycle-specific drug sensitivity, microenvironmental influences, and the presence of drug-tolerant persister cells.

Drug-tolerant persisters (DTPs) represent a crucial subset of tumor cells characterized by their slow growth kinetics and ability to survive initial drug treatment. Recent research has shown that these cells maintain their viability through distinct metabolic adaptations and epigenetic modifications [90]. The transition to and from this persister state appears to be dynamically regulated, allowing tumor cell populations to maintain a reservoir of potentially resistant cells.

The impact of spatial heterogeneity on tumor growth kinetics has gained increased attention through advanced imaging studies. Variations in nutrient availability, oxygen gradients, and mechanical stress across different regions of the tumor create distinct microenvironmental niches that influence both growth patterns and drug response [91]. These spatial variations can lead to the emergence of regional drug resistance through both genetic and nongenetic mechanisms.

Mathematical modelling of tumor growth kinetics has revealed complex patterns of clonal evolution during treatment. Studies utilizing integrative approaches combining single-cell sequencing, spatial transcriptomics, and computational modelling have demonstrated that resistance often emerges through the selective expansion of preexisting resistant subclones rather than the de novo acquisition of resistance mutations [92]. This understanding has important implications for therapeutic strategies, suggesting the need for early intervention with combination therapies.

The relationship between the tumor growth rate and immune system interactions has emerged as a critical factor in resistance development. Recent research has shown that rapidly growing tumors can overwhelm immune responses, whereas slower-growing tumors may establish more effective immune evasion mechanisms [93]. This dynamic interplay influences both natural tumor progression and the response to immunotherapy.

The role of cancer stem cells (CSCs) in tumor growth kinetics has been further elucidated through lineage-tracing studies. These cells typically exhibit slower proliferation rates but maintain the ability to regenerate tumor heterogeneity, contributing to treatment resistance and disease recurrence [94]. Understanding the unique growth characteristics of CSCs has led to the development of targeted strategies to overcome their inherent resistance mechanisms.

Technological advances in the real-time monitoring of tumor growth have provided new insights into the temporal dynamics of resistance development. Studies using continuous measurements of tumor burden during treatment have revealed complex patterns of adaptation, including periods of apparent stability followed by rapid progression [95]. These observations highlight the importance of temporal considerations in therapeutic decision-making.

The impact of circadian rhythms on tumor growth kinetics and drug resistance has gained recognition through chronobiology studies. Research has demonstrated that both tumor cell proliferation and drug metabolism exhibit significant temporal variations, suggesting the potential importance of treatment timing in preventing resistance development [96]. This understanding has led to the exploration of chronotherapy approaches in cancer treatment.

A comprehensive analysis of cancer drug resistance mechanisms requires a systematic review of various drug classes and their specific resistance mechanisms. Table 1 provides an overview of major resistance mechanisms occurring in commonly used chemotherapeutic agents and targeted therapies. These mechanisms encompass both cellular adaptations and changes in the tumor microenvironment. Additionally, Table 2 presents a detailed analysis of resistance mechanisms specific to different targeted therapies, including secondary mutations, activation of alternative pathways, and microenvironmental modifications. 

## 13. Increased Drug Efflux

P-glycoprotein (P-gp/ABCB1) remains the most extensively studied drug efflux pump, with recent structural analyses revealing new insights into its substrate recognition and transport mechanisms. Advanced cryo-EM studies have identified specific conformational changes during the drug transport cycle, providing crucial information for the development of new generation inhibitors [125]. Furthermore, novel posttranslational modifications of P-gp that regulate its trafficking and stability in resistant cancer cells have been discovered.

Multidrug resistance-associated protein 1 (MRP1/ABCC1) has emerged as another critical mediator of drug resistance, particularly in relation to newly developed targeted therapies. Recent research has demonstrated that MRP1 can efficiently export various conjugated metabolites of kinase inhibitors, contributing to resistance in multiple cancer types [126]. Importantly, new studies have revealed that MRP1 expression can be rapidly induced in response to therapeutic stress through specific transcriptional programs.

Breast cancer resistance protein (BCRP/ABCG2) has gained increasing attention because of its role in resistance to molecular targeted therapies. Advanced proteomics analyses have identified novel interaction partners that regulate BCRP activity and localization in cancer cells [127]. These interactions provide new therapeutic opportunities for overcoming BCRP-mediated drug resistance.

Recent research has revealed complex regulatory networks controlling ABC transporter expression and function. Single-cell RNA sequencing studies have revealed that resistant cancer cells can dynamically modulate their ABC transporter expression profiles in response to treatment [128]. This adaptability allows cancer cells to maintain effective drug efflux even under varying therapeutic pressures.

The role of the tumor microenvironment in regulating drug efflux has become increasingly recognized. New findings demonstrate that cancer-associated fibroblasts can induce ABC transporter expression in tumor cells through specific signalling pathways, creating a protective environment that promotes drug resistance [129]. Additionally, hypoxia has been shown to increase ABC transporter activity through both HIF-dependent and HIF-independent mechanisms.

Emerging evidence suggests that ABC transporters can function as part of larger molecular complexes that integrate drug efflux with other cellular processes. Recent studies have revealed direct interactions between ABC transporters and various signalling molecules, suggesting that these proteins may also function as signalling hubs in cancer cells [130]. This expanded understanding of ABC transporter function provides new opportunities for therapeutic intervention.

## 14. Challenge of Oncogenes and Drug Resistance

The relationship between oncogenes and drug resistance represents a complex and dynamic interplay that significantly influences cancer treatment outcomes. Recent advances in molecular profiling and functional genomics have revealed intricate mechanisms through which oncogenic signalling contributes to therapeutic resistance and disease progression.

Oncogenic bypass mechanisms have emerged as critical components of resistance development. Advanced genomic studies have demonstrated that cancer cells can activate alternative oncogenic pathways when primary drivers are inhibited [131]. This adaptive response often involves complex signalling networks and cross-talk between multiple oncogenic pathways, making therapeutic targeting particularly challenging. Recent research has identified previously unknown compensatory mechanisms, including novel protein-protein interactions and unexpected pathway convergence points.

The concept of oncogene addiction has been refined through single-cell analyses and real-time monitoring studies. While cancer cells may initially depend on specific oncogenic drivers, research has revealed remarkable plasticity in this context [132]. Advanced studies using CRISPR-based screens have identified mechanisms through which cells can rapidly shift their oncogenic dependencies, particularly under therapeutic pressure. This adaptability often involves complex epigenetic reprogramming and changes in cellular metabolism.

The amplification and overexpression of oncogenes represent classical resistance mechanisms that have gained new understanding through modern genomic approaches. Recent studies using spatial transcriptomics have revealed that oncogene amplification can occur heterogeneously within tumors, creating distinct resistance niches [133]. Furthermore, research has shown that the timing and pattern of these amplification events can significantly influence treatment outcomes and the evolution of resistance.

The role of oncogenic feedback loops in drug resistance has been extensively investigated via systems biology approaches. New research has revealed that oncogenic signalling networks can establish complex feedback mechanisms that maintain cell survival even in the presence of targeted inhibitors [134]. These adaptive responses often involve rapid posttranslational modifications and changes in protein localization, allowing cancer cells to sustain critical survival signals.

Mutations in oncogenes leading to drug resistance have been comprehensively mapped via next-generation sequencing technologies. Recent studies have identified novel mutation patterns that emerge under therapeutic pressure, including previously unrecognized variants that confer resistance to targeted therapies [135]. Importantly, research has shown that these mutations can preexist in small subpopulations of cells or arise de novo during treatment.

The interaction between oncogenic signalling and the tumor microenvironment has emerged as a crucial factor in resistance development. Studies have demonstrated that oncogene-driven changes in the microenvironment can create protective niches that promote drug resistance [136]. This includes alterations in the extracellular matrix composition, the metabolic reprogramming of stromal cells, and the modulation of immune responses.

The epigenetic regulation of oncogene expression has gained increased attention as a mechanism of drug resistance. Recent research has revealed that dynamic changes in chromatin structure and DNA methylation patterns can rapidly modulate oncogene expression in response to therapy [137]. These epigenetic adaptations often provide cancer cells with remarkable flexibility in maintaining oncogenic signalling despite therapeutic intervention.

The role of noncoding RNAs in oncogene-mediated resistance has been extensively characterized through RNA sequencing studies. Research has identified complex networks of microRNAs and long noncoding RNAs that regulate oncogene expression and contribute to resistance development [138]. These regulatory networks often provide cancer cells with additional layers of adaptive response to therapeutic challenges.

Posttranslational modifications of oncoproteins have emerged as critical regulators of drug resistance. Advanced proteomics studies have revealed novel modifications that can alter protein function, stability, and localization in ways that promote resistance [139]. Understanding these modifications has led to new therapeutic strategies aimed at disrupting specific protein modifications rather than directly targeting oncoproteins.

## 15. Tumor Microenvironment and the Immune System

The tumor microenvironment (TME) and its complex interactions with the immune system are critical determinants of the development of treatment resistance. Recent advances in single-cell technologies, spatial transcriptomics, and advanced imaging have revealed unprecedented insights into how these dynamic interactions influence therapeutic outcomes and drive resistance mechanisms.

### 15.1. Cellular Components of the Resistant TME

The composition of the TME significantly evolves during treatment, with distinct cellular populations emerging to promote resistance. Recent single-cell analyses have revealed complex networks of interactions between cancer cells and various stromal components [140]. This dynamic interplay involves multiple cellular populations that collectively contribute to treatment resistance through diverse mechanisms.

Cancer-associated fibroblasts (CAFs) have emerged as key mediators of resistance, with distinct subpopulations providing specific protective functions. Advanced lineage tracing studies have identified specialized CAF subtypes that secrete specific factors that promote drug resistance and immune evasion [141]. Recent research has revealed that CAFs can be classified into several distinct subtypes, including myCAFs (myofibroblastic CAFs), iCAFs (inflammatory CAFs), and apCAFs (antigen-presenting CAFs), each with unique roles in treatment resistance. These CAF populations demonstrate remarkable plasticity and can transition between different functional states in response to therapy-induced stress [142].

Myeloid-derived suppressor cells (MDSCs) and tumor-associated macrophages (TAMs) play crucial roles in establishing an immunosuppressive microenvironment. Recent research has demonstrated that these cells undergo significant phenotypic and functional changes during treatment, resulting in the adoption of more immunosuppressive characteristics [143]. The polarization of TAMs toward an M2-like phenotype is particularly important in resistance development, as these cells produce factors that promote tumor growth, angiogenesis, and matrix remodelling while suppressing antitumor immunity [144].

### 15.2. Metabolic Reprogramming in the TME

The metabolic landscape of the TME significantly influences treatment resistance through multiple interconnected mechanisms. Advanced metabolomics studies have revealed complex nutrient competition between tumor cells and immune cells, leading to the functional impairment of antitumor immunity [145]. This competition extends beyond glucose metabolism to include amino acids, fatty acids, and other key metabolites. Treatment-resistant tumors often establish metabolic symbioses with stromal cells, creating localized zones of altered metabolism that support cancer cell survival and immune evasion [146].

Recent research has identified novel mechanisms through which hypoxia in the TME promotes resistance. Spatial transcriptomics analyses have revealed distinct hypoxic niches that harbor resistant cell populations and suppress immune function [147]. These hypoxic regions trigger complex adaptive responses, including the activation of HIF-1α-dependent pathways, metabolic reprogramming, and autophagy induction. Furthermore, hypoxia promotes the selection of more aggressive and stem-like cancer cells while simultaneously impairing immune cell function and drug delivery [142].

### 15.3. Extracellular Matrix Remodelling

The extracellular matrix (ECM) undergoes significant remodelling during treatment, contributing to resistance development through multiple mechanisms. Advanced proteomics studies have identified specific ECM modifications that create physical and biochemical barriers to drug delivery and immune cell infiltration [148].

These alterations include the following:

1. Increased ECM density and stiffness [149]:
  ○ Enhanced collagen crosslinking
  ○ Modified fibronectin assembly
  ○ Altered proteoglycan composition
2. Biochemical Modifications [150]:
  ○ Posttranslational modifications of ECM proteins
  ○ Release of bioactive ECM fragments
  ○ Modified growth factor sequestration
3. Mechanical Signal Transduction [151]:
  ○ Altered mechanotransduction pathways
  ○ Modified cellular adhesion dynamics
  ○ Changes in matrix metalloproteinase activity.

Influence of the microbiota on the TME

Recent studies have also highlighted the critical role of the microbiota in modulating the TME and treatment resistance [152]. Influences of the microbiota:

○ Immune system function and composition
○ Metabolic profiles within the TME
○ Drug metabolism and efficacy
○ Inflammatory responses
○ Barrier function and tissue homeostasis

Therapeutic Implications

Understanding these complex interactions has led to the development of novel therapeutic strategies targeting specific components of the TME [153,154]:

○ CAF-targeted therapies
○ ECM-modifying agents
○ Metabolic intervention approaches
○ Immunomodulatory strategies
○ Microbiota manipulation

## 16. Immune System Dynamics in Treatment Resistance

The complex interactions between the immune system and cancer cells during treatment are critical determinants of the development of therapeutic resistance. Understanding these dynamics has become increasingly important as immunotherapy continues to emerge as a fundamental approach in cancer treatment [155]. Recent advances in single-cell technologies and multiparameter immune profiling have revealed unprecedented insights into the mechanisms through which tumors evade immune surveillance and develop resistance to various therapeutic interventions [156].

T-cell exhaustion has emerged as a fundamental mechanism of immune evasion in treatment-resistant tumors [157]. This process involves the progressive loss of effector functions and the sequential upregulation of multiple inhibitory receptors, including PD-1, LAG-3, TIM-3, and TIGIT. Single-cell profiling has revealed distinct molecular programs associated with different stages of T-cell exhaustion, with specific transcriptional and epigenetic signatures marking the transition from reversible to irreversible dysfunction states [158]. These exhausted T cells exhibit profound metabolic alterations, including impaired mitochondrial function and altered glucose metabolism, which significantly impact their ability to mount effective antitumor responses [159].

The antigen presentation machinery undergoes significant modifications during the development of treatment resistance [160]. Tumors employ multiple strategies to disrupt effective antigen presentation, including the downregulation of MHC class I molecules, the impairment of antigen processing pathways, and the selective loss of tumor-specific antigens. These alterations often occur heterogeneously within the tumor, creating distinct zones of immune privilege that contribute to treatment resistance [161]. Recent studies have demonstrated that defects in the antigen presentation machinery can be both inherent and acquired, with specific genetic and epigenetic mechanisms driving these changes during treatment [162].

Myeloid cell plasticity plays a crucial role in establishing and maintaining an immunosuppressive microenvironment [163]. Tumor-associated macrophages and myeloid-derived suppressor cells undergo significant phenotypic and functional changes during treatment and adopt more immunosuppressive characteristics. These cells express high levels of inhibitory molecules and produce various factors that directly suppress T-cell function and promote tumor growth. The recruitment and activation of these immunosuppressive myeloid cells are often driven by specific chemokine networks that become dysregulated during the development of treatment resistance [164].

The metabolic landscape of the tumor microenvironment significantly influences immune cell function and contributes to treatment resistance [145]. Competition for essential nutrients between tumor cells and immune cells creates metabolic zones that impair effective antitumor immunity. Additionally, the accumulation of immunosuppressive metabolites and altered oxygen availability further compromise immune cell function [165]. Recent research has revealed complex metabolic interactions between different cell populations within the tumor microenvironment, highlighting the importance of metabolic reprogramming in resistance development.

Cytokine and chemokine networks undergo extensive remodelling during treatment resistance [166]. These changes include an increased production of immunosuppressive cytokines such as IL-10 and TGF-β, altered chemokine gradients affecting immune cell trafficking, and the dysregulation of interferon signalling pathways [158]. The resulting inflammatory environment often promotes tumor growth while suppressing effective antitumor immune responses. Understanding these complex signalling networks has led to the development of novel therapeutic strategies aimed at reversing the immunosuppressive TME [167].

The role of regulatory T cells (Tregs) in treatment resistance has gained increased attention [168]. These cells undergo selective expansion and functional enhancement during treatment, contributing to the maintenance of an immunosuppressive environment. Recent studies have identified specific mechanisms through which Tregs suppress antitumor immunity and promote treatment resistance. These insights have led to the development of targeted approaches to modulate Treg function while preserving beneficial immune responses [169].

Novel immune evasion mechanisms continue to be discovered, including the formation of physical barriers through a modified extracellular matrix, the development of specialized immune-excluded niches, and the altered circadian regulation of immune responses [161,164]. Additionally, the identification of new immune checkpoint pathways has expanded our understanding of the complex regulatory networks controlling antitumor immunity [167].

Understanding these immune system dynamics has important therapeutic implications [165]. Novel approaches targeting T-cell exhaustion, enhancing antigen presentation, reprogramming myeloid cells, and modulating metabolic pathways are being developed [156]. These strategies, often used in combination with existing therapies, show promise in overcoming treatment resistance. Furthermore, the identification of biomarkers associated with specific immune evasion mechanisms may allow for more personalized therapeutic approaches [170].

## 17. Contribution to Understanding Drug Resistance

This comprehensive analysis has significantly advanced our understanding of cancer drug resistance in several key areas:

1. Mechanistic Insights
  ○ Identification of novel resistance pathways and their interactions
  ○ Understanding resistance evolution during treatment
  ○ Recognition of the role of tumor heterogeneity
  ○ Characterization of adaptive response mechanisms
2. Clinical Applications
  ○ Development of improved resistance monitoring strategies
  ○ Identification of novel therapeutic targets
  ○ Optimization of combination therapy approaches
  ○ Enhancement of personalized treatment selection
3. Treatment innovation
  ○ Design of mechanism-specific inhibitors
  ○ Development of resistance prevention strategies
  ○ Implementation of adaptive treatment protocols
  ○ Integration of biomarker-guided approaches

## 18. Conclusions and Future Perspectives

Recent advances in understanding cancer drug resistance have revealed its complex, multilevel nature, integrating genetic, epigenetic, and microenvironmental factors. This comprehensive analysis identified key aspects of resistance development:

1. Molecular Complexity
  ○ Dynamic interactions between multiple resistance pathways
  ○ Adaptive responses to therapeutic pressure
  ○ Integration of tumor heterogeneity with microenvironmental factors
2. Therapeutic Implications
  ○ Need for mechanism-specific combination strategies
  ○ Importance of biomarker-guided treatment selection
  ○ Development of resistance monitoring approaches
  ○ Integration of immunological and metabolic targeting
3. Research Priorities
  ○ Investigation of resistance mechanism interactions
  ○ Development of real-time monitoring technologies
  ○ Enhancement of predictive biomarkers
  ○ Implementation of adaptive therapeutic strategies

Success in overcoming drug resistance will require an integrated approach combining the following:

○ Mechanistic understanding of resistance pathways
○ Development of targeted therapeutic strategies
○ Implementation of personalized medicine approaches
○ Integration of real-time resistance monitoring

These advancements promise to improve cancer treatment outcomes through more effective, personalized therapeutic strategies that can anticipate and overcome resistance mechanisms.

## Figures and Tables

**Table 1:** Resistance mechanisms against commonly used chemotherapeutic agents and targeted therapies.

Drug Group	Types of Cancer	Molecular Target	Mechanisms of Resistance	Ref.
Antimetabolites (5-FU, methotrexate, gemcitabine, cytarabine)	Breast cancer, colon cancer, pancreatic cancer, stomach cancer, head and neck cancer, ovarian cancer, lymphomas, leukemias	Thymidylate synthase and DNA synthesis	Increased target expression (thymidylate synthase) Changes in DNA methylation (MLH1, DPYD) Activation of survival pathways (ERBB, PI3K/AKT) Enhanced anti-apoptotic signalling Metabolic reprogramming Alternative splicing of target genes Increased stem cell population	[97,98]
Platinum compounds (cisplatin, oxaliplatin, carboplatin)	Ovarian cancer, testicular cancer, sarcomas, lymphomas, NSCLC, bladder cancer	DNA	Reduced cellular uptake (reduced CTR1) Increased efflux (increased ATP7A/B) Enhanced DNA repair Epigenetic modifications Alternative RNA splicing Activation of bypass pathways Altered mitochondrial function	[99,100]
Topoisomerase I/II inhibitors (irinotecan, doxorubicin, etoposide)	Colon cancer, SCLC, Kaposi’s sarcoma, Ewing’s sarcoma, lymphomas, leukemias, glioma	Topoisomerase I/II	Enhanced drug efflux (ABCB1, ABCG2) Target modifications Activation of DNA damage response Metabolic adaptations Interactions with tumor microenvironment Expression of alternative isoforms	[101,102]
Drugs acting on microtubules (paclitaxel, vinorelbine)	Lung cancer, ovarian cancer, breast cancer, head and neck cancer, Kaposi’s sarcoma	Tubulin	Tubulin mutations and isoform switching Increased expression of ABC transporters Altered posttranslational modifications Activation of EMT Metabolic reprogramming Altered microtubule dynamics	[103,104]
Targeted therapies (TKI, monoclonal antibodies)	Various solid and hematologic tumors	Specific molecular targets (EGFR, ALK, BRAF)	Secondary target mutations Activation of bypass pathways Phenotypic transformation Tumor heterogeneity Alternative splicing Cell line plasticity	[105,106]
Immunotherapies (checkpoint inhibitors, CAR-T)	Many types of cancer	Immune system	Loss of target antigen Immune editing T-cell exhaustion Activation of alternative checkpoints Metabolic competition Altered tumor microenvironment	[107,108]

**Table 2:** Mechanisms of resistance to molecularly targeted drugs.

Targeted Therapy	Type of Cancer	Molecular Target	Mechanisms of Resistance	Ref.
BCR-ABL tyrosine kinase inhibitors (Imatinib, Dasatinib, Nilotinib, Ponatinib, Asciminib)	CML, ALL, GIST	BCR-ABL1, KIT, PDGFRα	Kinase domain point mutations (T315I, F317L) BCR-ABL1 amplification Activation of alternative pathways (SRC, STAT3) Epigenetic modifications Changes in cellular metabolism Leukemia stem cell resistance	[109,110]
HER2 inhibitors (Trastuzumab, Pertuzumab, T-DXd)	HER2+ Breast Cancer, Stomach Cancer	ERBB2/HER2	Loss of PTEN Activating mutations of PIK3CA Alternative forms of HER2 (p95HER2) Activation of bypass pathways (IGF1R, MET) Modulation of the microenvironment Heterogeneity of HER2 expression Resistance to drug-antibody conjugate	[111,112]
EGFR inhibitors (Gefitinib, Osimertinib, Cetuximab)	NSCLC, colon cancer, head and neck cancer	EGFR	Secondary mutations (T790M, C797S) EMT transformation MET/HER2 amplification KRAS mutations Epigenetic reprogramming Metabolic changes Activation of the histone deacetylase pathway	[113,114]
BRAF inhibitors (Vemurafenib, Dabrafenib, Encorafenib)	Melanoma, NSCLC	BRAF-V600E	MAPK pathway reactivation NRAS/MEK mutations Activation of alternative pathways (PI3K/AKT) Increased BRAF expression Microenvironment modifications Tumor heterogeneity Metabolic adaptations	[115,116]
ALK inhibitors (Crizotinib, Alectinib, Lorlatinib)	NSCLC	EML4-ALK	ALK secondary mutations Activation of alternative kinases Changes in cellular metabolism Gene rearrangements Epigenetic modifications Cellular plasticity	[117,118]
Proteasome inhibitors (Bortezomib, Carfilzomib)	Multiple myeloma, mantle cell lymphoma	Proteasome	Binding site mutations Anti-apoptotic mechanisms Metabolic adaptations Changes in autophagy Endoplasmic reticulum stress Microenvironmental modifications	[119,120]
Angiogenesis inhibitors (Bevacizumab, Ramucirumab)	Various solid tumors	VEGF/VEGFR	Alternative angiogenic pathways Hypoxia and autophagy Increased stem cell population Modulation of the microenvironment Metabolic adaptation Recruitment of immunosuppressive cells	[121,122]
CDK4/6 inhibitors (Palbociclib, Ribociclib)	HR+/HER2-Breast Cancer	CDK4/6	Loss of Rb Activation of PI3K/AKT pathway Cyclin E amplification Epigenetic modifications Metabolic changes Cellular plasticity	[123,124]
